# Effects of plyometric training on health-related physical fitness in untrained participants: a systematic review and meta-analysis

**DOI:** 10.1038/s41598-024-61905-7

**Published:** 2024-05-17

**Authors:** Nuannuan Deng, Kim Geok Soh, Borhannudin Bin Abdullah, Dandan Huang, Fan Xu, Marrium Bashir, Dong Zhang

**Affiliations:** 1 Department of Sports Studies, Faculty of Educational Studies, Universiti Putra Malaysia, Selangor, Malaysia; 2 College of Physical Education, Chongqing University, Chongqing, China

**Keywords:** Plyometric exercise, Stretch–shortening cycle, Physical fitness, Cardiorespiratory fitness, Muscular fitness, Physiology, Health care

## Abstract

Plyometric training (PT) is an effective training method for improving physical fitness among trained individuals; however, its impact on health-related physical fitness in untrained participants remains ambiguous. Therefore, this meta-analysis aimed to evaluate the effects of PT on health-related physical fitness among untrained participants. Six electronic databases (PubMed, CINAHL Plus, MEDLINE Complete, Web of Science Core Collection, SCOPUS, and SPORTDiscus) were systematically searched until March 2024. We included controlled trials that examined the effects of PT on health-related physical fitness indices in untrained participants. Twenty-one studies were eligible, including a total of 1263 participants. Our analyses revealed small to moderate effects of PT on body mass index, muscular strength, cardiorespiratory fitness, and flexibility (ES = 0.27–0.61; all p > 0.05). However, no significant effects were detected for body fat percentage and lean mass (ES = 0.21–0.41; all p > 0.05). In conclusion, the findings suggest that PT may be potentially effective in improving health-related physical fitness indices (i.e., body mass index, muscular strength, cardiorespiratory fitness, and flexibility) in untrained participants. However, the results should be interpreted cautiously due to data limitations in some fitness variables.

## Introduction

Physical fitness is characterized by the capacity to engage in daily activities with energy and alertness, without excessive fatigue, while still enjoying leisure pursuits and responding effectively to unexpected emergencies^1^. Health-related physical fitness pertains to the specific aspects of physical fitness closely linked to overall health and well-being^2^. The level of health-related physical fitness is a predictive factor for cardiovascular and metabolic diseases, as well as the overall risk of mortality across one’s lifespan^3,4^. The components of health-related physical fitness include body composition, muscular strength, muscular endurance, flexibility, and cardiorespiratory fitness^5^. Numerous studies have consistently demonstrated that adequate health-related fitness is linked to a reduced risk of disease and an improved quality of life. For example, insufficient cardiorespiratory fitness has been correlated with an increased incidence of hypertension^6^, cardiovascular diseases^7^, and mental health disorders such as depression^8^. Likewise, sub-optimal body composition (e.g., low muscle mass and high adipose tissue levels) is associated with significant chronic ailments, such as cancers, type 2 diabetes, and mortality^9^.

Plyometric training (PT) is a highly favored training approach frequently recommended by researchers as an effective method for enhancing various aspects of physical performance^10–13^. Conceptually, PT is characterized by the utilization of the stretch–shortening cycle (SSC), which occurs during the transition from a swift eccentric muscle contraction (deceleration phase) to a quick concentric muscle contraction (acceleration phase)^14^. SSC movements take advantage of the elastic properties of connective tissue and muscle fibers, enabling muscles to store elastic energy during the deceleration phase and release it during the acceleration phase to augment muscular force and power production^15,16^. Moreover, PT induces numerous favorable adaptations in musculoskeletal and neural systems, muscle function and performance of healthy individuals^17^. In this sense, by enhancing the SSC and related neuro-mechanical mechanisms, PT can potentially improve human performance.

In the literature, many systematic reviews and meta-analyses have demonstrated that PT enhances physical fitness. For example, PT has proven beneficial in improving the physical fitness of athletes across a diverse range of sports, including team sports^18^, water sports^19^, racket sports^20^, and combat sports^21^. Moreover, several meta-analyses have confirmed that PT can improve physical fitness in both healthy young individuals and adults^22–25^. A meta-analysis by de Villarreal et al.^26^ emphasized that PT significantly increases strength performance. Similarly, Ramirez-Campillo et al.^27^ identified PT as an effective and safe form of exercise for enhancing body composition. However, these research efforts have mainly focused on athletic groups or a mix of trained and untrained subjects. The impact of PT may differ significantly depending on various characteristics of the subjects, such as their level of training, age, gender, or familiarity with plyometric exercises^26^. Consequently, the effectiveness and feasibility of PT in enhancing overall health-related physical fitness among untrained individuals remain underexplored, with a notable lack of comprehensive reviews on this topic.

Historically, plyometric exercises have typically demanded a significant degree of neuromuscular control and a substantial level of strength, often indicated by requirements such as a back squat of at least 150% of one’s body mass^28^. This has generated uncertainty among practitioners regarding the safety and feasibility of plyometric exercises for untrained individuals. These concerns arise from the fact that untrained populations typically lack the fundamental neuromuscular control and strength levels traditionally considered prerequisites for athletes before engaging in high-intensity PT^29^. On the contrary, some experts argue that basic competency in bodyweight movements should suffice before gradually introducing simple plyometric exercises into a training regimen^30^. In line with this viewpoint, PT has been successfully integrated into the routines of very elderly individuals over the age of 75^31^ and young children under ten years of age^32,33^, with no reported injuries or adverse events.

Despite a growing number of experimental trials investigating the impact of PT on untrained individuals, the effects on health-related physical fitness seem inconsistent. For example, while some researchers^34,35^ have documented improvements in cardiorespiratory fitness after PT, others have found no such improvements^36,37^. Moreover, some studies^38,39^ have identified positive effects of PT on body composition, such as reductions in body fat percentage, while other studies^37,40^ have reported no positive impact. Hence, it is urgent for investigators to find an appropriate way to address the existing conflicting results. Conducting a systematic review and meta-analysis, which involves systematically collecting and screening relevant studies and rigorously assessing the quality of the included research, represents the highest level of evidence in evidence-based practice^41^. Currently, no systematic review or meta-analysis has been published regarding the effect of PT interventions on overall health-related physical fitness in untrained populations. Therefore, the primary objective of this systematic review and meta-analysis was to identify and rigorously assess the existing research findings derived from collected data and to pool the results of publications in a meta-analysis. By doing so, we intend to provide a well-informed conclusion regarding the impact of PT on health-related physical fitness in untrained participants.

## Materials and methods

The present review is reported following the updated PRISMA statement^42^, and the review protocol has been registered in the PROSPERO (identifier CRD42023473050).

### Search strategy

We conducted a comprehensive literature search by accessing six electronic databases: PubMed, CINAHL Plus, MEDLINE Complete, Web of Science Core Collection, SCOPUS, and SPORTDiscus, from their inception to March 20, 2024. Specific combinations of terms were tailored for each of these databases: (“plyometric training” OR “ballistic training” OR “jump training” OR “plyometric exercise*” OR “power training” OR “stretch–shortening cycle”) AND (“physical fitness” OR “body composition” OR “body weight status” OR “body mass” OR BMI OR “body fat” OR “cardiorespiratory fitness” OR “cardiorespiratory endurance” OR “muscular fitness” OR “musculoskeletal fitness” OR “muscular strength” OR “muscular endurance” OR “flexibility”). Moreover, a thorough manual search was conducted on both Google Scholar and the reference lists of all selected papers to ensure that no relevant publications were missed. The search string for each database can be found in Supplementary Material Appendix 1.

### Selection criteria

The inclusion criteria following the PICOS framework^43^ were applied as follows: (a) untrained participants who did not engage in any systematic training or competitive sport, without restrictions on sex and age; (b) a PT intervention lasting more than two weeks, including lower body exercises (e.g., jumping, hopping, skipping) and/or upper body exercises (e.g., medicine ball exercises, push-ups) utilizing the SSC. Studies that incorporated combined training (e.g., PT and balance training) were included in the analysis when the control group underwent the same training regimen, except the PT component; (c) a control group; (d) at least one measure of health-related physical fitness parameters; (e) randomized controlled or non-randomized controlled design.

Studies were excluded if they (a) involved injured individuals (e.g., ankle sprain); (b) had interventions lasting shorter than two weeks; (c) did not provide adequate results (e.g., mean and standard deviation); (d) tested the effects of PT without a control group; (e) training interventions that do not include PT or training interventions where PT programs make up less than 50% of the total training load when combined with other training methods (e.g., heavy resistance training), and (f) were conducted in languages other than English. Because of translation difficulties and most of the literature on PT is in English^44^, only English language studies were included.

### Risk of bias in individual studies and certainty of evidence

The risk of bias in each selected randomized controlled trial (RCTs) was evaluated using the updated Cochrane risk of bias assessment for randomized trials (RoB-2)^45^. For non-randomized controlled trials (non-RCTs), the Risk Of Bias In Non-randomized Research of Interventions (ROBINS-I) tool was employed^46^. The certainty of evidence was analyzed and summarized following the guidelines outlined in the GRADE handbook^47^. Two research team members independently (ND, KGS) evaluated the risk of bias for each selected trial.

### Data extraction

The data items were common metrics of health-related physical fitness, including (a) body composition (e.g., body mass index (BMI), body fat percentage, and lean mass), (b) muscular strength (e.g., handgrip), (c) muscular endurance (e.g., sit-ups), (d) cardiorespiratory fitness (e.g., maximal oxygen uptake (VO_2_max)), and (e) flexibility (e.g., sit and reach). Apart from the mentioned data elements, descriptive characteristics of the PT interventions (e.g., length, frequency) and the participants (e.g., sex, age) were extracted, and adverse effects were recorded. To conduct the meta-analysis, we chose original articles that provided data suitable for calculation and utilized consistent outcome measures.

### Statistical analyses

When at least three trials provided sufficient data to calculate the effect size (ES), a meta-analysis was conducted^19,48^. Mean and standard deviation data from pre- and post-intervention measures were used to compute ESs for performance outcomes in both the PT and control groups (i.e., Hedges’ g). The data were standardized using the post-intervention standard deviation values, and a random-effects model was employed to account for variances across trials that could potentially affect the effects of PT^49,50^. The values of ES were accompanied by 95% confidence intervals (CIs), and the calculated ES values were interpreted using the following scale: ES < 0.2 was considered trivial, 0.2–0.6 was classified as small, > 0.6–1.2 was considered moderate, > 1.2–2.0 was categorized as large, > 2.0–4.0 was considered very large, and ES > 4.0 was considered extremely large^51^. In trials involving multiple intervention groups, the sample size of the control group was split proportionately so that all subjects could be compared^52^. In cases where authors did not submit adequate data (in graphics or were missing), we attempted to contact the corresponding authors. If the authors did not reply to our requests or could not supply the relevant data, the investigation’s findings were dropped from the study. However, if data were presented in a figure but no numerical data was included in the tables or supplementary material, we utilized the Graph Digitizer software (Digitizelt, Germany) to extract the relevant data from the graphs or figures^53^. We evaluated study heterogeneity using I^2^ statistics. Values below 25% indicated low heterogeneity, 25–75% suggested moderate heterogeneity, and above 75% reflected high heterogeneity^54^. The extended Egger’s test was employed to assess the publication bias risk in the studies^55^. When bias was detected, the trim and fill method was utilized. Stratification of the meta-analyses was performed for each of these factors, and a threshold of *p* < 0.05 was utilized as the significance level to determine statistical significance. The Comprehensive Meta-Analysis software (Version 3.0; Biostat, Englewood, NJ, USA) was used for all analyses. In addition, if it was not feasible to statistically pool the data, the findings were presented in a narrative format.

## Results

### Study selection

**Figure 1:**
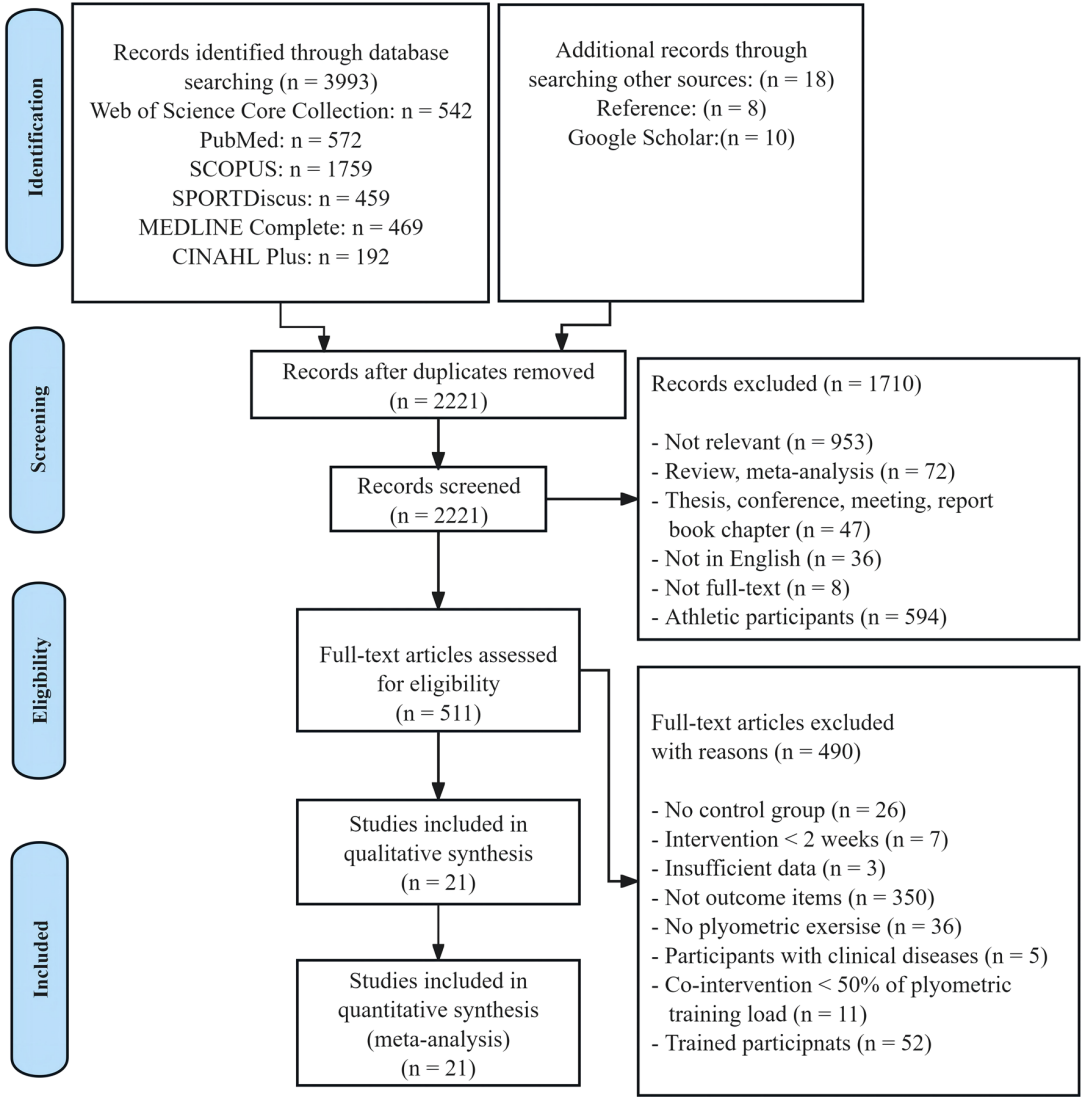
PRISMA flow diagram.

As shown in Fig. 1, the databases yielded a total of 3993 documents, with an additional 18 publications obtained via references and Google Scholar. After manually removing duplicates, there were 2221 unique records remaining. These records’ titles and abstracts were evaluated, yielding 511 publications acceptable for full-text examination. Following a careful evaluation of all the texts, 490 documents were removed, leaving 21 studies that met all of the criteria set for the systematic review and meta-analysis.

### Risk of bias in individual studies and certainty of evidence

**Figure 2:**
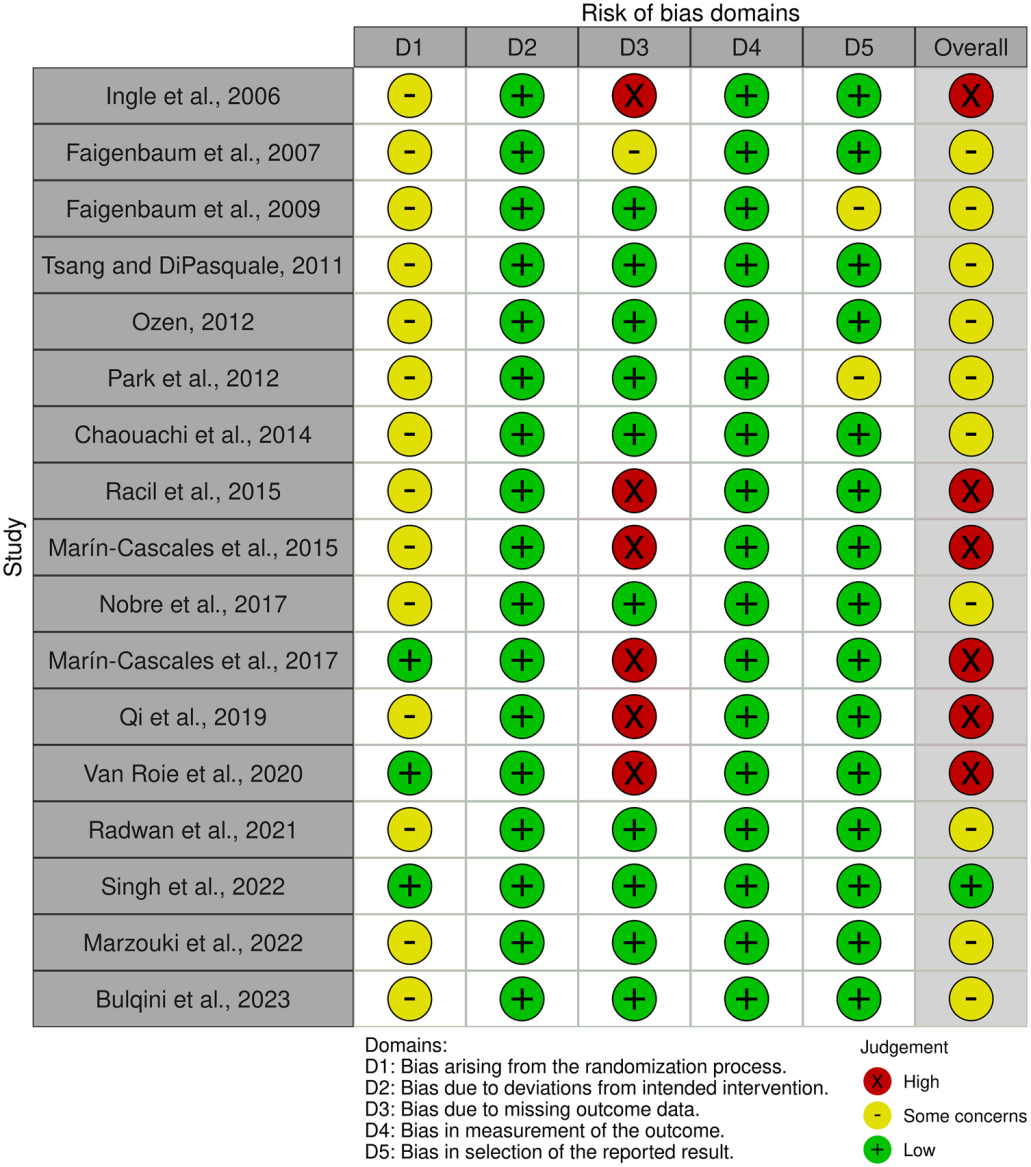
RoB-2 assessments. Created using Robvis tool.

**Figure 3:**
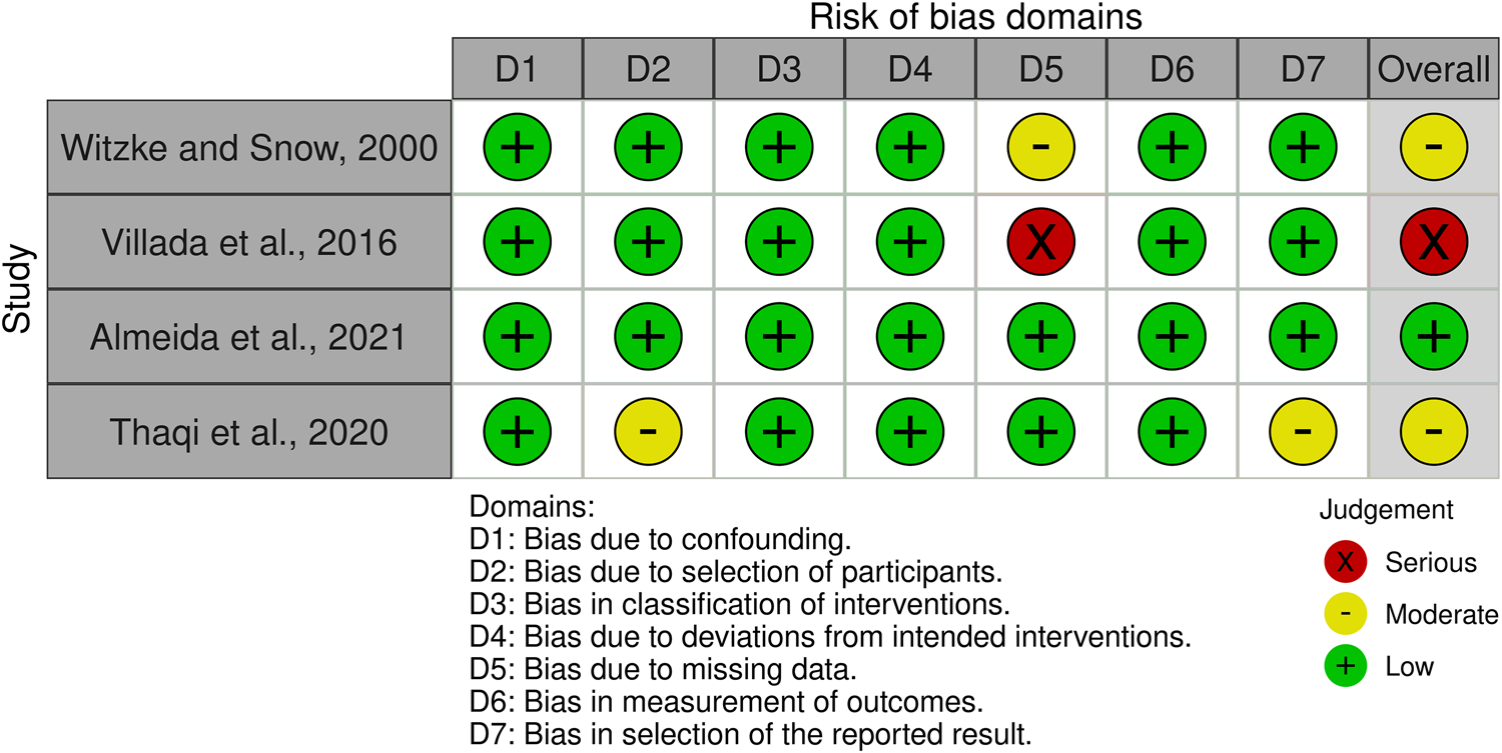
ROBINS-I assessments. Created using Robvis tool.

RoB-2 assessments were conducted on 17 RCTs^34,35,37–40,56–66^, while ROBINS-I assessments were employed for four non-RCTs^36,67–69^. Out of these trials, 12 exhibited an overall moderate risk of bias or some concerns^35,37,40,56–60,64,66,67,69^, seven noted a high (or serious) risk of bias^34,38,39,61–63,68^, and only two showed a low risk of bias^36,65^, as illustrated in Figs. 2 and 3. Figure 2 presents the results of the RoB-2 assessments. Among these RCTs, only three reported their randomization sequence generation method, which involved either stratified block randomization or online randomization software (www.randomizer.org)^61,63,65^. In contrast, the remaining eleven articles did not comprehensively describe their randomized procedures. In six RCTs, a high risk of bias was identified due to missing data, which was linked to a dropout rate exceeding 15%^34,38,39,61–63^. A visual depiction of the findings of ROBINS-I evaluations is shown in Fig. 3. A non-RCT study identified a moderate risk of bias due to an approximately 10% dropout rate and concerns regarding the selection of reported outcomes^69^. Another study^68^ revealed a high risk of bias because of a 25% dropout rate. Additionally, one trial had a moderate risk of bias in selecting study participants^67^.

Table 1 shows the GRADE analysis results. The GRADE analyses found very low to moderate levels of certainty in the evidence supporting the outcomes.

**Table 1:** GRADE analyses.

Outcomes	Certainty assessment					Number of participants and studies	Certainty of evidence (GRADE)
	Risk of bias	Inconsistency	Indirectness	Imprecision	Risk of publication bias		
Body composition (body mass index) follow-up: range 12 to 22 weeks	Serious^a^	Serious^b^	Not serious	Serious^c^	Not serious	369 (4 studies)	⊕◯◯◯VERY LOW
Body composition (body fat percentage) follow-up: range 6 to 36 weeks	Serious^a^	Serious^b^	Not serious	Serious^c^	Not serious	341 (8 studies)	⊕◯◯◯VERY LOW
Body composition (lean mass) follow-up: range 12 to 36 weeks	Serious^a^	Not serious	Not serious	Serious^c^	Not serious	149 (4 studies)	⊕⊕◯◯LOW
Muscular strength follow-up: range 4 to 36 weeks	Serious^a^	Not serious	Not serious	Not serious	Not serious	865 (15 studies)	⊕⊕⊕◯MODERATE
Cardiorespiratory fitness follow-up: range 4 to 12 weeks	Serious^a^	Not serious	Not serious	Serious^c^	Serious^d^	493 (6 studies)	⊕⊕◯◯LOW
Flexibility follow-up: range 6 to 12 weeks	Serious^a^	Not serious	Not serious	Serious^c^	Not serious	276 (4 studies)	⊕⊕◯◯LOW

GRADE Working Group grades of evidence High certainty: we are very confident that the true effect lies close to that of the estimate of the effect. Moderate certainty: we are moderately confident in the effect estimate: the true effect is likely to be close to the estimate of the effect, but there is a possibility that it is substantially different. Low certainty: our confidence in the effect estimate is limited: the true effect may be substantially different from the estimate of the effect. Very low certainty: we have very little confidence in the effect estimate, the true effect is likely to be substantially different from the estimate of the effect.

*GRADE* Grading of Recommendations Assessment, Development and Evaluation.

^a^Downgraded by one level due to high or some concerns risk of bias.

^b^Downgraded by one level due to the substantial heterogeneity (I^2^ ≥ 50%).

^c^Downgraded by one level, as < 400 participants were available for a comparison or there was an unclear direction of the effects. Downgraded by two levels in case of imprecision based on both assessed points.

^d^Downgraded by one level due to the significant of Egger’s test (p < 0.05).

### Study characteristics

Table 2 provides a detailed overview of the participants’ characteristics and PT programs employed in the included studies. Supplementary Material Appendix 2 contains the data used in the meta-analyses. Publications were released between 2000 and 2023. The included studies involved a collective participation of 1263 subjects, comprising 417 females and 846 males. The sample sizes within the study groups ranged from 15 to 220 subjects, with participants’ ages spanning from 7 to 69.5 years. The majority of studies exclusively recruited either males (n = 11)^36–38,40,56,60,62,63,65,66,69^ or females (n = 7)^34,39,58,61,64,67,68^, while other three studies included both female and male participants^35,57,59^. Nine studies provided information on the effects of PT interventions on body composition^34,37–40,61,62,67,68^, 15 on muscular strength^36–39,57–64,66,67,69^, six on cardiorespiratory fitness^34–37,57,65^, five on flexibility^36,37,56,57,64^, and one on muscular endurance^69^. The length of PT intervention varied from 4 to 36 weeks, with most trials opting for a 12-week intervention period (n = 8). The planned training sessions ranged from 12 to 108, with weekly training sessions set at either two or three. The duration of each session varied from 15 to 90 min.

**Table 2:** Characteristics of the studies examined in the present review.

Study	Design	Population characteristics			Intervention	Type of exercise	Test (s)	Outcome (s)
		Sex	n	Age				
Ingle et al. (2006)^38^	RCT	M	54	12.3 ± 0.3 yrs	Freq: 3 times/week Time: 60-75 min Length: 12 weeks	EG: Plyometric exercises + resistance training CG: Habitual levels of physical activity	Body composition (body fat%), muscular strength (chest pass)	Body fat% ↑, chest pass ↑
Faigenbaum et al. (2007)^56^	RCT	M	27	EG: 13.6 ± 0.7 yrs CG: 13.4 ± 0.9 yrs	Freq: 2 times/week Time: 50 min Length: 6 weeks	EG: Plyometric + resistance training CG: Resistance training	Flexibility (SAR)	SAR ↑
Faigenbaum et al. (2009)^57^	RCT	Mixed	74 (44 M/30F)	8–1 yrs	Freq: 2 times/week Time: 10–15 min Length: 9 weeks	EG: Plyometric training CG: Physical education sessions	Muscular strength (push-up), cardiorespiratory fitness (half-mile run), flexibility (SAR)	Push-up ↑, half-mile run ↑, SAR ↔
Tsang and DiPasquale (2011)^58^	RCT	F	25	20.3 ± 1.9 yrs	Freq: 3 times/week Time: 45–60 min Length: 6 weeks	EG: Plyometric training CG: Daily activities	Muscle strength (knee flexion)	Knee extensor ↑
Ozen (2012)^40^	RCT	M	19	EG: 21.2 ± 2.3 yrs CG: 21.4 ± 2.1 yrs	Freq: 2–3 times/ week Time: NR Length: 6 weeks	EG: Plyometric Training CG: Routine activities	Body composition (body fat %)	Body fat % ↔
Park et al. (2012)^59^	RCT	Mixed	31 (14 M/17F)	EG: 76.7 ± 8.6 yrs CG: 77.0 ± 7.9 yrs	Freq: 5 times/week Time: 30 min Length: 4 weeks	EG: Jump training + therapeutic exercise CG: Therapeutic exercise	Muscular strength (knee extension)	Knee extensor ↑
Chaouachi et al. (2014)^60^	RCT	M	42	12–15 yrs	Freq: 3 times/week Time: 60–90 min Length: 8 weeks	EG1: Plyometric training EG2: Balance + plyometric training CG: Routine activities	Muscular strength (leg press)	Leg press ↑
Racil et al. (2015)^34^	RCT	F Obesity	68	16.6 ± 1.3 yrs	Freq: 3 times/week Time: NR Length: 12 weeks	EG: Plyometric exercise + HIIT CG: HIIT	Body composition (Body fat %, MBI, lean mass), cardiorespiratory fitness (VO_2_max)	Body fat % ↑, MBI ↑, lean mass ↑, VO_2_max ↑
Marín-Cascales et al. (2015)^39^	RCT	F	38	59.8 ± 6.2 yrs	Freq: 3 times/week Time: NR Length: 12 weeks	EG1: Vibration training EG2: Jump training + aerobic activity CG: Daily routines	Body composition (lean mass, body fat%) Muscular strength (knee extension)	Knee extension ↑, body fat% ↑, lean mass ↑
Nobre et al. (2017)^37^	RCT	M Overweight/obese	59	7–9 yrs	Freq: 2 times/week Time: 20 min Length: 12 weeks	EG: Plyometric Training CG: Regular physical activity	Body composition (BMI, body fat%), muscle strength (handgrip), flexibility (SAR), cardiorespiratory fitness (VO_2_max)	BMI ↔ , body fat% ↔ , handgrip ↑, SAR ↑, VO_2_max ↔
Marín-Cascales et al. (2017)^61^	RCT	F	38	60.0 ± 6.3 yrs	Freq: 3 times/week Time: NR Length: 24 weeks	EG1: Vibration training EG2: Jumps + aerobic activity CG: No intervention	Body composition (lean mass, body fat%) Muscular strength (knee extension)	Knee extension ↑, body fat% ↑, Lean mass ↔
Qi et al. (2019)^62^	RCT	M	46	8–12 yrs	Freq: 2 times/week Time: 60 min Length: 12 weeks	EG: Plyometric training + resistance CG: Physical education sessions	Body composition (BMI, lean body mass), muscular strength (biceps curl)	BMI ↔ , lean body mass ↔ , Biceps curl ↑
Van Roie et al. (2020)^63^	RCT	M	40	69.5 ± 3.9 yrs	Freq: 3 times/week Time: 35 min Length: 12 weeks	EG1: Plyometric training EG2: Resistance training CG: Walking	Muscular strength (leg press)	Leg press ↑
Radwan et al. (2021)^64^	RCT	F	40	9–11 yrs	Freq: 2 times/week Time: 20 min Length: 9 weeks	EG: Plyometric training CG: Routine activities	Muscle strength (knee extension), flexibility (SAR)	Knee extension ↑, SAR ↑
Singh et al. (2022)^65^	RCT	M	75	20.1 ± 1.7 yrs	Freq: 2 times/week Time: 15–60 min Length: 9 weeks	EG1: Plyometric training + endurance running (outdoor) EG2: Plyometric training + endurance running (treadmill) CG: Regular physical activity	Cardiorespiratory fitness (cooper test)	Cooper test ↑
Marzouki et al. (2022)^35^ study 1	RCT	M	60	8–11.5 yrs	Freq: 2 times/week Time: NR Length: 4 weeks	EG1: Plyometric training (clay surface) EG2: Plyometric training (dry surface) CG: Physical education sessions	Cardiorespiratory fitness (VO_2_max)	VO_2_max ↑
Marzouki et al. (2022)^35^ study 2	RCT	F	60	8–11.5 yrs	Freq: 2 times/week Time: NR Length: 4 weeks	EG1: Plyometric training (clay surface) EG2: Plyometric training (dry surface) CG: Physical education sessions	Cardiorespiratory fitness (VO_2_max)	VO_2_max ↑
Bulqini et al. (2023)^66^	RCT	M	30	20.10 ± 1.32 yrs	Freq: 3 times/week Time: NR Length: 6 weeks	EG1: Plyometric knee tuck jump training EG2: Plyometric hurdle jump training CG: Routine activities	Muscle strength (leg muscle strength)	Leg muscle strength ↑
Witzke and Snow (2000)^67^	Non-RCT	F	56	14.6 ± 0.5 yrs	Freq: 3 times/week Time: 30–45 min Length: 36 weeks	EG: Plyometric exercises + resistance training CG: Habitual physical activity	Body composition (body fat %, lean mass), muscular strength (knee extension)	Body fat % ↔ , knee extension ↔ , lean mass ↔
Villada et al. (2016)^68^	Non-RCT	F	45	59.45 ± 6.43 yrs	Freq: 3 times/week Time: NR Length: 22 weeks	EG1: Jump training EG2: Concurrent jump + machines training CG: Routine activities	Body composition (BMI, body fat %)	BMI ↔ , body fat % ↑
Thaqi et al. (2020)^69^	Non-RCT	M	220	16 yrs ± 6 months	Freq: 2 times/week Time: 60 min Length: 12 weeks	EG: Plyometric training CG: Routine activities	Body composition (BMI), muscle strength (PU30s), muscle endurance (SUP30s)	BMI ↔ , SUP30s ↑, PU30s ↑
Almeida et al. (2021)^36^	Non-RCT	M	116	7–9 yrs	Freq: 2 times/week Time: 20 min Length: 12 weeks	EG: Plyometric training CG: Routine activities	Muscle strength (handgrip), cardiorespiratory fitness (VO_2_max), flexibility (SAR)	handgrip ↑, VO_2_max ↔ , SAR ↑

*RCT* randomized controlled trial, *EG* experimental group, *CG* control group, *NR* not reported, *yrs* years, *M* male, *F* female, *Freq* frequency, *VO2max* maximal oxygen consumption, *MBI* body mass index, *HIIT* high-intensity interval training, ↑ significant within-group improvement, ↔ non-significant within-group, *SAR* sit-and-reach, *SUP30s* sit-ups in 30 s, *PU30s* push-ups in 30 s.

### Synthesis of the results

#### Body composition

**Figure 4:**
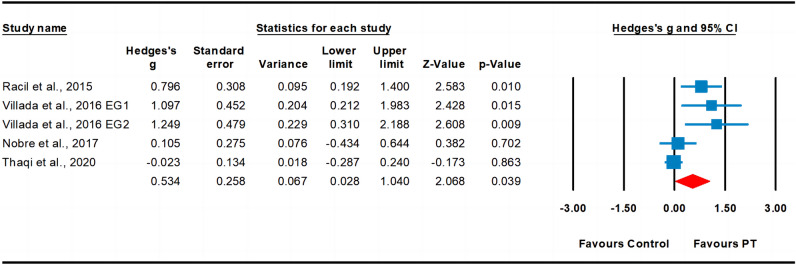
Forest plot and effect sizes for plyometric training (PT) compared with controls for body mass index. *EG* experimental group.

Five studies assessed BMI, involving six experimental groups and five control groups (pooled n = 369). The results indicated a small effect of PT on BMI (ES = 0.46; 95% CI − 0.09 to 1.01; *p* = 0.099). A high heterogeneity (I^2^ = 91.83%) was observed, and the Egger’s test indicated *p* = 0.920. After we removed one study^62^ from the analysis, we found a significant moderate effect in favor of PT while heterogeneity decreased (ES = 0.53; 95% CI 0.03–1.04; *p* < 0.039, I^2^ = 73.91%; Fig. 4).

**Figure 5:**
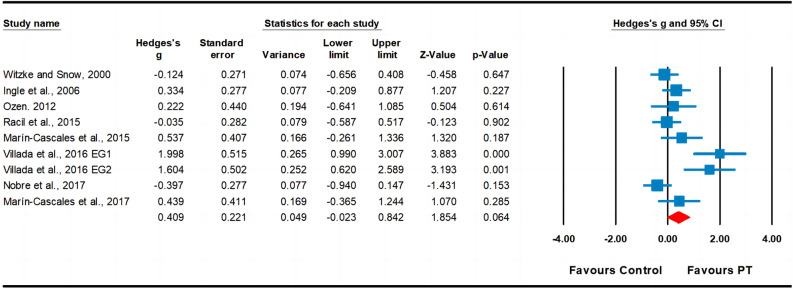
Forest plot and effect sizes for plyometric training (PT) compared with controls for body fat percentage. *EG* experimental group.

Regarding body fat percentage, data from eight studies were analyzed, including nine experimental groups and eight control groups (pooled n = 341). The results indicated a small effect of PT on body fat percentage (ES = 0.41; 95% CI − 0.02 to 0.84; *p* = 0.064; Fig. 5). A moderate heterogeneity (I^2^ = 71.38%) was observed, and the Egger’s test indicated *p* = 0.005. Since publication bias for body fat percentage was detected, we applied the trim and fill method; however, the ES remained unchanged.

**Figure 6:**
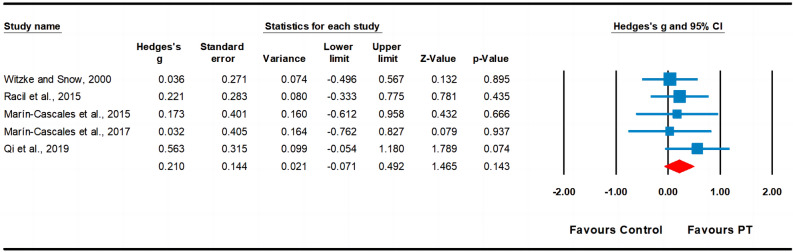
Forest plot and effect sizes for plyometric training (PT) compared with controls for lean mass.

Regarding lean mass, data from five studies were analyzed, including five experimental groups and five control groups (pooled n = 149). The results indicated a small effect of PT on lean mass (ES = 0.21; 95% CI − 0.07 to 0.49; *p* = 0.143; Fig. 6). A low heterogeneity (I^2^ = 0.00%) was observed, and the Egger’s test indicated *p* = 0.951.

#### Muscular strength

**Figure 7:**
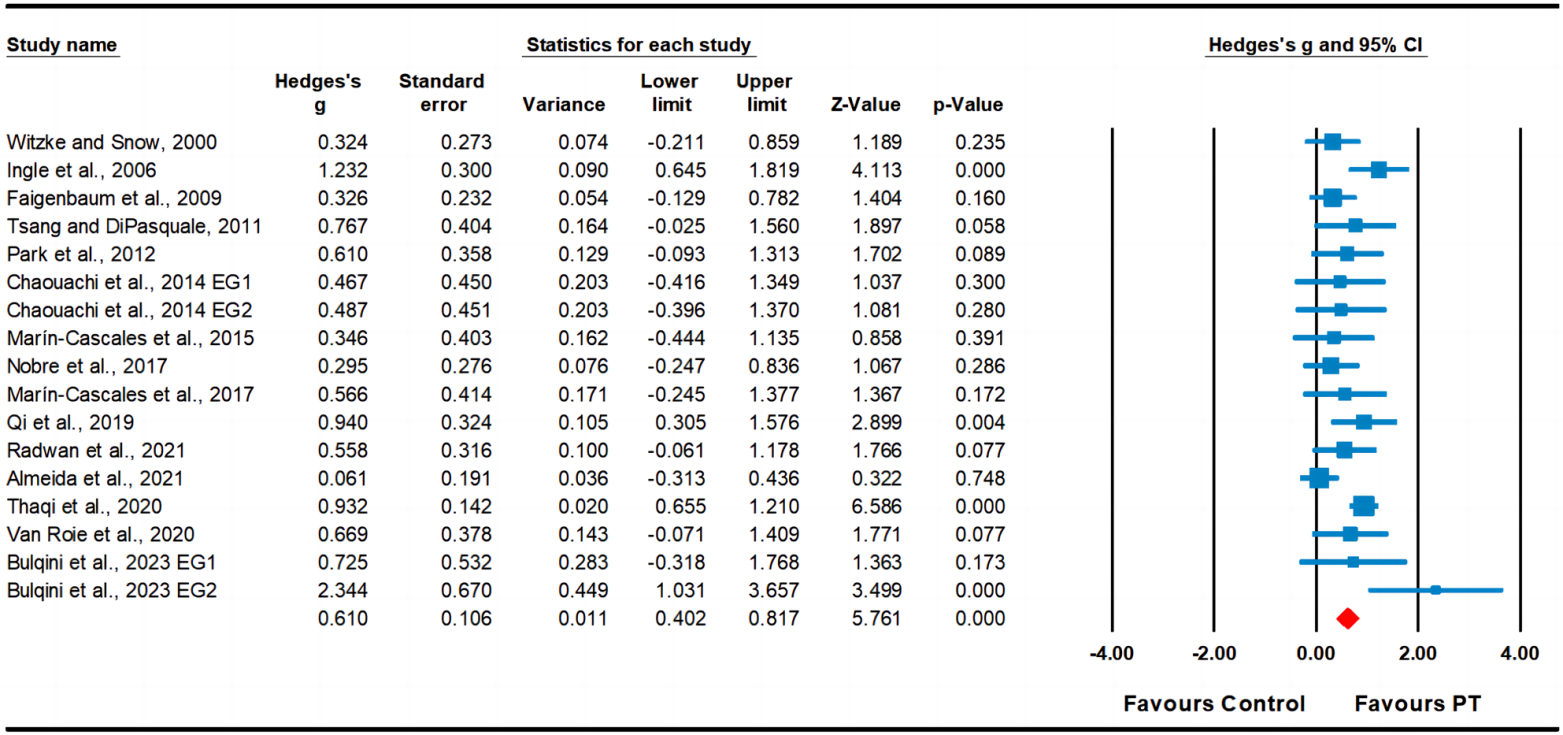
Forest plot and effect sizes for plyometric training (PT) compared with controls for muscular strength. *EG* experimental group.

Fifteen studies assessed muscular strength, involving seventeen experimental groups and fifteen control groups (pooled n = 865). The results indicated a moderate effect of PT on muscular strength (ES = 0.61; 95% CI 0.40–0.82; *p* < 0.001; Fig. 7). A moderate heterogeneity (I^2^ = 47.08%) was observed, and the Egger’s test indicated p = 0.669.

#### Cardiorespiratory fitness

**Figure 8:**
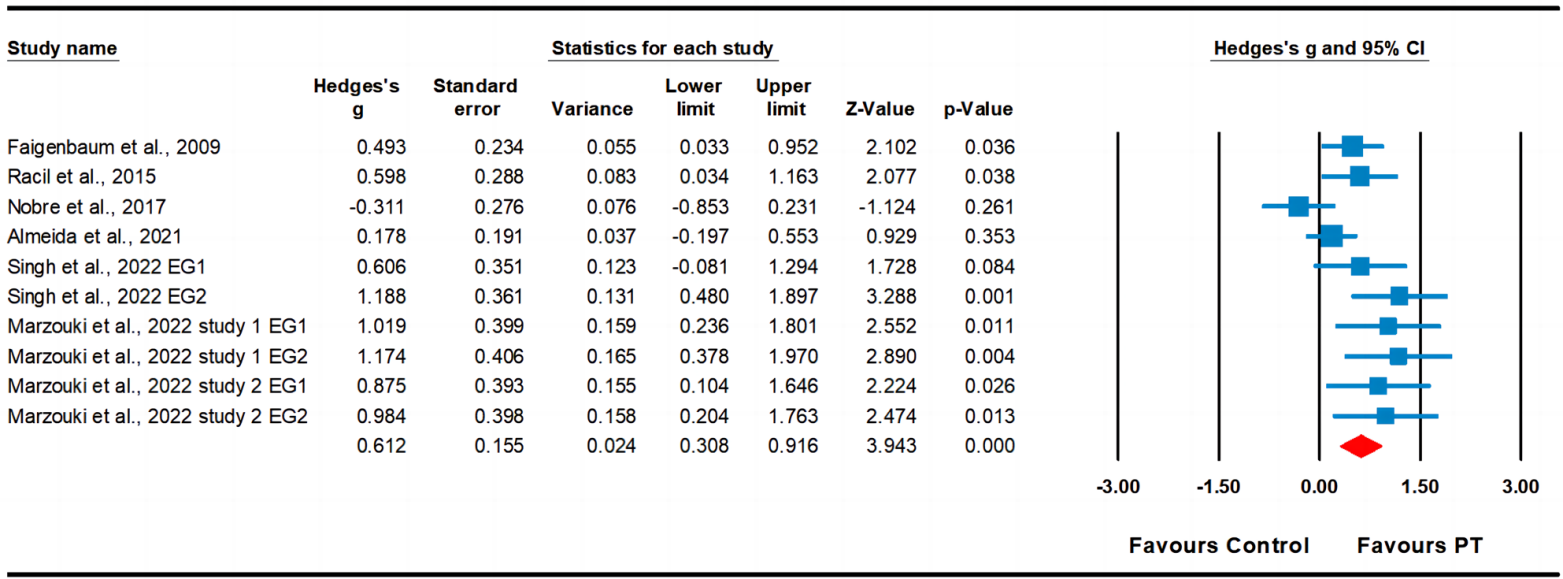
Forest plot and effect sizes for plyometric training (PT) compared with controls for cardiorespiratory fitness. *EG* experimental group.

Six studies assessed cardiorespiratory fitness, involving ten experimental groups and seven control groups (pooled n = 493). The results indicated a moderate effect of PT on cardiorespiratory fitness (ES = 0.61; 95% CI 0.31–0.92; *p* < 0.001; Fig. 8). A moderate heterogeneity (I^2^ = 59.28%) was observed, and the Egger’s test indicated *p* = 0.016. After the trim and fill method, the adjusted values indicated a point estimate of ES = 0.44 (95% CI 0.14–0.73).

#### Flexibility

**Figure 9:**
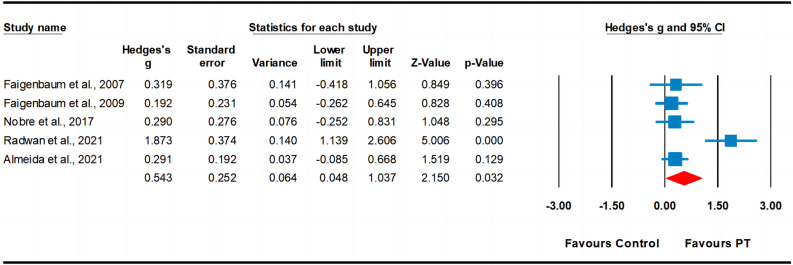
Forest plot and effect sizes for plyometric training (PT) compared with controls for flexibility.

Five studies assessed flexibility, involving five experimental groups and five control groups (pooled n = 276). The results indicated a moderate effect of PT on flexibility (ES = 0.54; 95% CI 0.05–1.04; *p* = 0.032). A high heterogeneity (I^2^ = 76.19%) was observed, and the Egger’s test indicated *p* = 0.274. After we removed one study^64^ from the analysis, although the significant effect of PT remained (ES = 0.27; 95% CI 0.03–0.51; *p* < 0.001; Fig. 9), the heterogeneity was reduced to 0.00%.

#### Muscular endurance

Due to the limited availability of studies, muscular endurance could not be included in the meta-analysis. Thus, the certainty of the evidence was automatically rated as very low. A study assessed the effect of PT on muscular endurance among 220 male high school students. The results revealed that the muscular endurance of adolescents significantly improved after 12 weeks of PT^69^.

#### Adverse effects

In a study conducted by Van Roie et al.^63^, which focused on older men aged 68–80 years, out of the 14 participants who initially joined the PT group, three individuals (one in week 5 and two in week 6) discontinued their participation due to various issues. One participant dropped out due to knee pain, while the other two experienced gastrocnemius muscle strain during forward or sideways step-up exercises. Four participants reported knee pain and five experienced mild muscle soreness. Notably, only three participants in the PT group did not report any side effects over the 12 weeks. In three other studies^37,39,58^, a few participants were excluded due to health issues, but the authors emphasized that these health issues were unrelated to the PT program. In addition, Villada et al.^68^ reported no injuries during the training period, except for subjective fatigue and muscle aches during the first weeks of participation. Beyond these instances, no other studies included in this review documented any cases of soreness, fatigue, injuries, pain, or adverse effects caused by PT intervention.

## Discussion

This meta-analysis examined peer-reviewed research on the effects of PT vs. controls on health-related physical fitness outcomes in untrained individuals. These analyses indicate that PT can significantly improve MBI, muscular strength, cardiorespiratory fitness, and flexibility compared to control conditions. However, the effects of PT on body fat percentage and lean mass were not statistically significant. In addition, based on the available data, it cannot be determined whether PT can enhance muscular endurance. According to the GRADE assessment, the level of evidence for the evaluated outcomes ranged from very low to moderate.

### The effect of PT on body composition

Enhancing lean body mass and decreasing the percentage of body fat are advantageous health outcomes in addressing the increasing prevalence of obesity^70^. In the present review, body composition was evaluated using BMI, body fat percentage, and lean mass. The included studies measured the body composition parameters using skinfold calipers^37,68^ and bioelectrical impedance analysis^34,62^. Bioelectrical impedance analysis has gained popularity due to its affordability, portability, and ease of use^71^. However, some researchers have noted that bioelectrical impedance analysis might overestimate the body fat percentage in lean and athletic individuals, while it might underestimate this percentage in those who are heavier and have a higher body fat percentage^72^. Our meta-analysis revealed that PT positively affects BMI in untrained individuals; however, it does not show significant benefits in reducing body fat percentage or improving lean body mass. It seems that changes in body composition are the main elements that restore energy balance via increased energy expenditure^73^. The observed improvement in BMI can be attributed to the increased energy expenditure and muscle mass gains associated with PT^74,75^. However, the absence of alterations in body composition parameters (i.e., body fat percentage and lean body mass) aligns with findings from a prior meta-analysis investigating the impact of lower extremity PT on body composition^27^. Moreover, several studies focusing on athletes found no substantial changes in body composition. For example, Campo et al.^76^ determined that a 12-week PT program had no significant effects on body fat in female soccer players. Similarly, in the case of Aloui et al.^77^, no substantial changes in body fat percentage were observed following an eight-week PT regimen for handball players.

Some researchers have pointed out that the age range plays a significant role in developmental changes, where substantial increases in weight and height may lead to negligible changes in body composition^78^. Vetrovsky et al.^24^ propose that PT may not be the primary exercise choice if the goal is to effect changes in body composition. Still, it could be integrated into a periodized program to yield additional functional adaptations that may not be achieved through other types of exercise interventions. It is worth noting that a study by Racil et al.^34^ discovered that PT, when added to high-intensity interval training in overweight/obese females, led to a more substantial decrease in body fat in comparison to high-intensity interval training alone. Furthermore, several studies suggest combining exercise training with dietary adjustments leads to decreased body fat and increased lean mass^79–81^. Additionally, some investigators have claimed that aerobic exercise is the preferred method for reducing body fat and body mass. Concurrently, they recommend adopting a training program incorporating plyometric exercises to enhance lean body mass^82,83^. In this regard, researchers are encouraged to delve deeper into the dose-response relationships concerning PT variables, such as exercise type, intensity, and volume, or their combination with other strategies. This exploration is expected to improve our understanding of the relationship between PT and body composition measures, potentially enhancing the health benefits for untrained populations.

### The effect of PT on muscular strength

Muscular strength is the capacity to exert force under specified biomechanical conditions^84^. This physical attribute is essential, as it influences proficiency in executing various tasks, whether in sports or daily life^85^. The evaluation of muscular strength across different studies incorporated various measurement methods, including leg press, knee extension, handgrip strength, and push-ups. These tests evaluated strength in distinct muscle groups, which may explain the moderate level of heterogeneity observed in the analysis. Our findings indicate that positive effects on the muscular strength of untrained participants were observed after PT. These results align with the conclusions drawn from previous meta-analyses^9,26^. Strength increases resulting from PT are based on the physiological principle that stretching a muscle before contraction leads to greater force generation^26^. In short, PT-induced enhancements in muscular strength can be attributed to several neuromuscular adaptations, including (a) improved neural activation of agonist muscles, (b) modifications in single-fiber mechanics, (c) changes in muscle size and structure, and (d) alterations in muscle-tendon mechanical stiffness^14,16,86^. Furthermore, the gains in muscular strength following PT can also be attributed to muscle hypertrophy^87^. Additionally, increased body weight and height naturally occurring with age could be connected to improvements in muscular strength^88,89^. Given that most of the research in our review assessed muscular strength in children and adolescents, the results presented here can be considered a typical and expected response to training.

According to a systematic review, it appears that PT may positively impact muscular strength in older adults, although perhaps not to the same magnitude as resistance training^24^. In addition, resistance training is a popular method used to improve muscular strength^85,90^. In our analysis, research conducted by Van Roie et al.^63^ investigated the impact of PT compared to regular walking and reported favorable results. Besides, they observed similar improvements in muscular strength after 6 weeks of PT compared to conventional resistance exercises in older individuals. Nevertheless, older people who participate in PT may be at risk of injury (e.g., knee pain)^63^. There is a need for additional studies to determine the optimal training parameters (e.g., volume, intensity, frequency, and specific exercises) to enhance muscular strength gains and reduce injury risks among older individuals. Another study conducted by Faigenbaum et al.^56^ demonstrated that incorporating plyometric exercises into a resistance training program proved to be more effective than resistance training alone in enhancing muscular strength. Collectively, our findings suggest that PT could serve as a viable approach for improving muscular strength in untrained populations.

### The effect of PT on cardiorespiratory fitness

Cardiorespiratory fitness is commonly acknowledged as essential in preventing heart disease^91,92^. Improving cardiorespiratory fitness is strongly associated with overall health enhancements^93^. Findings from research conducted by Azmi et al.^94^ suggest that maintaining elevated levels of cardiorespiratory fitness in childhood is linked to maintaining a healthy BMI and reducing cardiometabolic risks in adolescence and adulthood. VO_2_max is widely accepted as the gold standard for assessing cardiorespiratory fitness^95^ and reflects the maximum rate of oxygen consumption and the physiological processes behind it^96^. Data on cardiovascular fitness, quantified through VO_2_max, were available in four of the included studies^34–37^. Our meta-analysis identified positive effects of PT on cardiorespiratory fitness. Among the six studies included, two^34,37^ examined changes in cardiorespiratory fitness among overweight or obese individuals. Racil et al.^34^ found that 12 weeks of PT coupled with high-intensity intermittent training significantly improved cardiorespiratory fitness in obese female adolescents. Conversely, Nobre et al.^37^ observed no significant effects of 12 weeks of PT on cardiorespiratory fitness in obese boys. Another study^65^ demonstrated that a nine-week PT program improved cardiorespiratory fitness in adult males, while Almeida et al.^36^ reported that a 12-week PT program did not lead to enhanced cardiorespiratory fitness in children. Interestingly, a previous study reported that the training surface can influence the outcomes of PT^97^. However, one study included in this review, involving plyometric exercises on different surfaces (sand and firm surfaces), suggested that the type of surface did not significantly affect the cardiorespiratory fitness changes induced by the training^35^.

In the athletic literature, Mazurek et al.^10^ conducted a study involving young handball players and noted a significant increase in cardiorespiratory fitness after 5 weeks of PT. Similarly, Lum et al.^94^ recruited endurance runners and observed a significant improvement in cardiorespiratory fitness following 6 weeks of PT. Ramirez-Campillo et al.^98^ compared the performance of young soccer players before and after an 8-week PT intervention and identified noticeable enhancements in cardiorespiratory fitness. Notably, it appears that PT yields significant changes in cardiorespiratory fitness among both athletic and non-athletic populations. The enhancement of cardiorespiratory fitness could be attributed to the heightened energetic cost associated with performing PT^99^. Moreover, aerobic training may improve markers of cardiorespiratory fitness (e.g., blood volume, cardiac output) through its beneficial effects on central adaptations. Furthermore, improved cardiorespiratory fitness might result from an increase in mitochondrial content, an activation of adenosine monophosphate-activated protein kinase, and an elevated maximal activity of citrate synthase^100–102^. Overall, the findings from our review supported the notion that PT can effectively enhance cardiorespiratory fitness among untrained individuals.

### The effect of PT on flexibility

Flexibility enables a joint to move easily through its entire range of motion and also encompasses a muscular component related to muscle length^5^. This attribute of flexibility plays a role in injury prevention, posture enhancement, alleviating back pain, and reducing muscle soreness^103^. Five studies evaluated flexibility using the sit-and-reach tests, which are likely the most widely utilized methods for assessing hamstring and lower back flexibility^104^. Our findings indicate that PT improves flexibility performance in untrained participants. The improvements in flexibility within the PT group can be attributed to increased excitability of neuromuscular receptors in muscles, tendons, joints, and ligaments^16^. Specifically, the improved flexibility might be explained by the potential reductions in stiffness within the muscle–tendon complex and similar alterations in the elastic properties of the surrounding joint structures^86,105^. Moreover, PT can induce changes in the elastic properties of both muscle and connective tissues through motor unit recruitment and the frequency of neural firing. This, in turn, potentiates the reflex arc and leads to increased neuromuscular adaptation^17^. Furthermore, it reduces the Golgi tendon organs’ responsiveness to excessive tensile loads within the muscles, enabling enhanced stretching of the muscular elastic components^16^.

These findings align with some studies that have observed statistically significant improvements in flexibility among athletes. De Villarreal et al.^106^ reported that a 7-week period of PT resulted in increased flexibility among high-school basketball players. Similarly, da Silva et al.^107^ confirmed the beneficial impact of a 4-week PT regimen on improving flexibility in female Futsal athletes. Furthermore, it is also plausible that participants who frequently engaged in static or dynamic stretching before plyometric exercises may have experienced a beneficial impact on flexibility. Stretching routines increase muscle temperature, activate the nervous system, improve intramuscular coordination, and enhance muscle elasticity^108^. Overall, the improvement of flexibility in untrained participants after PT is an important finding, because a deficit in this health-related fitness component has been linked to muscle and joint injuries in the lower extremities.

### The effect of PT on muscular endurance

Greater muscular endurance can lower the risk of falling and related injuries^109,110^. However, only one study^69^ in the current review examined the impact of 12 weeks of PT on muscular endurance in adolescents and reported significant enhancements in this aspect of fitness. This trial^69^ measured muscular endurance using the sit-up test, which is a safe, cost-effective, and applicable method for evaluating core muscular endurance in both females and males^111^. As far as our knowledge extends, very limited information is available on this finding, making further elaboration and discussion somewhat challenging. Consequently, we can speculate that PT may enhance the properties of the SSC within the muscles, resulting in improved musculotendinous and neural unit performance, ultimately facilitating maximal force generation in the shortest time^14^. Such improvements could be associated with enhancements in muscular endurance. However, due to the scarcity of studies addressing this point, further research is imperative before conclusive recommendations can be made.

### Limitations

There are a few limitations worth noting in this review. Firstly, due to the scarcity of research for each programming parameter, we did not conduct subgroup analyses on PT variables (e.g., frequency, length, and total sessions) for physical fitness performance measures. Moreover, apart from studies on muscular strength (n = 15), only a small number of studies included in the meta-analysis assess the effects of PT on other fitness indices (n = 5–8). Secondly, none of the studies examined potential confounding factors that could influence the relationship between physical exercise and outcomes (i.e., health-related physical fitness), such as sleep behaviors and dietary habits. Thirdly, according to the GRADE evaluation, the level of certainty in the reported fitness outcomes ranged from very low to moderate, weakening the confidence of these estimates. Finally, the growth and maturation of young participants may affect the overall training effects^112^. Nevertheless, 11 studies included in this review recruited children or adolescents without providing information about their biological maturity. Despite the limitations mentioned above, our review offers a novel and noteworthy value to the current body of knowledge, shedding light on the efficacy of PT in enhancing health-related physical fitness in untrained populations.

## Practical applications

The findings of this study hold practical significance for physical education teachers and practitioners. Firstly, the analysis demonstrates that PT effectively enhances BMI, muscular strength, cardiorespiratory fitness, and flexibility in untrained participants. However, due to limited data, specific training variables for optimizing these aspects of physical fitness cannot be confidently recommended. Generally, the most important considerations when developing PT programs for BMI, muscular strength cardiorespiratory fitness, and flexibility were that effective programs included two to three sessions per week for a minimum of 4 weeks. Secondly, researchers are encouraged to undertake well-designed studies investigating the effects of PT on muscular endurance. The additional studies are crucial to validate further and reinforce the conclusions drawn in this analysis. Thirdly, the utilization of PT is a cost-effective alternative compared to other training strategies, as it requires no or little equipment. Typically, PT involves engaging in drills that utilize the participant’s body weight as a load^113^. This makes it a convenient and accessible training approach for untrained participants, allowing them to incorporate it into their daily routines easily.

## Conclusion

This meta-analysis revealed that PT may be potentially effective in improving health-related physical fitness indices (i.e., body mass index, muscular strength, cardiorespiratory fitness, and flexibility) in untrained participants. However, its effects on body fat percentage and lean muscle mass were not significant. Caution is warranted when interpreting these findings because of data limitations in some fitness variables. Future research should focus on conducting more high-quality studies on PT interventions for health-related physical fitness in untrained populations to provide more reliable evidence for practical applications in this field.

### Supplementary Information

Supplementary Information 1.

Supplementary Information 2.

## Data Availability

Data is provided within the manuscript or Supplementary Information files.

## Abbreviations

PT: Plyometric training
SSC: Stretch–shortening cycle
RCTs: Randomized controlled trial studies
Non-RCTs: Non-randomized controlled trials
PRISMA: Preferred reporting items for systematic reviews and meta-analyses
PROSPERO: International prospective register of systematic reviews
ROB-2: Risk of bias
ROBINS-I: In non-randomized research of interventions
GRADE: Grading of referrals, assessment, develop, and evaluations
CI: Confidence interval
ES: Effect size
