# Supplementary material for: Effects of plyometric training on health-related physical fitness in untrained participants: a systematic review and meta-analysis
Source: Sci Rep. 2024 May 17;14:11272. doi: 10.1038/s41598-024-61905-7 (PMC11101471; doi:10.1038/s41598-024-61905-7)
Supplement: Supplementary file 1 — Supplementary Information 1. [file 41598_2024_61905_MOESM1_ESM.docx]

**File 1. Detailed Search string**

**Search on 20 March 2024**

| **Databases** | **Search strategy** | **Results** |
| --- | --- | --- |
| PubMed | ("plyometric training" [Title/Abstract] OR "ballistic training" [Title/Abstract] OR "jump training" [Title/Abstract] OR "plyometric exercise*"[Title/Abstract] OR "power training" [Title/Abstract] OR "stretch-shortening cycle" [Title/Abstract]) AND ("physical fitness" [Title/Abstract] OR "body composition" [Title/Abstract] OR "body weight status" [Title/Abstract] OR "body mass" [Title/Abstract] OR BMI [Title/Abstract] OR "body fat" [Title/Abstract] OR "cardiorespiratory fitness" [Title/Abstract] OR "cardiorespiratory endurance" [Title/Abstract] OR "muscular fitness" [Title/Abstract] OR "musculoskeletal fitness" [Title/Abstract] OR "muscle strength" [Title/Abstract] OR "muscular endurance" [Title/Abstract] OR "flexibility" [Title/Abstract]) | 572 |
| Web of Science Core Collection | (AB=(“plyometric training” OR "ballistic training" OR “jump training” OR “plyometric exercise*” OR “power training” OR “stretch-shortening cycle”)) AND AB=(“physical fitness” OR “body composition” OR “body weight status” OR “body mass” OR BMI OR “body fat” OR “cardiorespiratory fitness” OR “cardiorespiratory endurance” OR “muscular fitness” OR “musculoskeletal fitness” OR “muscle strength” OR “muscular endurance” OR “flexibility”) | 542 |
| SPORTDicus | AB (“plyometric training” OR “ballistic training” OR “jump training” OR “plyometric exercise*” OR “power training” OR “stretch-shortening cycle”) AND AB (“physical fitness” OR “body composition” OR “body weight status” OR “body mass” OR BMI OR “body fat” OR “cardiorespiratory fitness” OR “cardiorespiratory endurance” OR “muscular fitness” OR “musculoskeletal fitness” OR “muscle strength” OR “muscular endurance” OR “flexibility”) | 459 |
| SCOPUS | (TITLE-ABS-KEY("plyometric training" OR "ballistic training" OR "jump training" OR "plyometric exercise*" OR "power training" OR "stretch-shortening cycle") AND TITLE-ABS-KEY(“physical fitness” OR “body composition” OR “body weight status” OR “body mass” OR BMI OR “body fat” OR “cardiorespiratory fitness” OR “cardiorespiratory endurance” OR “muscular fitness” OR “musculoskeletal fitness” OR “muscle strength” OR “muscular endurance” OR “flexibility”)) | 1759 |
| CINAHL Plus | AB (“plyometric training” OR “ballistic training” OR “jump training” OR “plyometric exercise*” OR “power training” OR “stretch-shortening cycle”) AND AB (“physical fitness” OR “body composition” OR “body weight status” OR “body mass” OR BMI OR “body fat” OR “cardiorespiratory fitness” OR “cardiorespiratory endurance” OR “muscular fitness” OR “musculoskeletal fitness” OR “muscle strength” OR “muscular endurance” OR “flexibility”) | 192 |
| MEDLINE Complete | AB (“plyometric training” OR “ballistic training” OR “jump training” OR “plyometric exercise*” OR “power training” OR “stretch-shortening cycle”) AND AB (“physical fitness” OR “body composition” OR “body weight status” OR “body mass” OR BMI OR “body fat” OR “cardiorespiratory fitness” OR “cardiorespiratory endurance” OR “muscular fitness” OR “musculoskeletal fitness” OR “muscle strength” OR “muscular endurance” OR “flexibility”) | 469 |
| Total |  | 3993 |
