# Supplementary material for: Effects of plyometric training on health-related physical fitness in untrained participants: a systematic review and meta-analysis
Source: Sci Rep. 2024 May 17;14:11272. doi: 10.1038/s41598-024-61905-7 (PMC11101471; doi:10.1038/s41598-024-61905-7)
Supplement: Supplementary file 2 — Supplementary Information 2. [file 41598_2024_61905_MOESM2_ESM.docx]

| **File 2** | **The data used for meta-analyses** | | | |  | | | | | |  | | |
| --- | --- | --- | --- | --- | --- | --- | --- | --- | --- | --- | --- | --- | --- |
| **Reference** | **Test** | **EG**  **(pre-test)** | | | **EG**  **(post-test)** | | | **CG**  **(pre-test)** | | | **CG**  **(post-test)** | | |
|  |  | **Mean** | **SD** | **n** | **Mean** | **SD** | **n** | **Mean** | **SD** | **n** | **Mean** | **SD** | **n** |
| Ingle et al., 2006 | Muscular strength (bench press) | 14.5 | 2.9 | 33 | 19.6 | 4.6 | 33 | 13.3 | 1.7 | 21 | 13.7 | 1.7 | 21 |
|  | Body composition (body fat%) | 22.6 | 6.1 | 33 | 21.0 | 6.1 | 33 | 22.2 | 3.9 | 21 | 22.4 | 3.7 | 21 |
| Faigenbaum et al., 2007 | Flexibility (SAR) | 18.4 | 8.5 | 13 | 21.2 | 8.0 | 13 | 20.5 | 8.9 | 14 | 25.6 | 5.9 | 14 |
| Faigenbaum et al., 2009 | Muscular strength (push-up) | 4.6 | 5.6 | 40 | 8.7 | 9.5 | 40 | 9.7 | 8.5 | 34 | 10.8 | 8.6 | 34 |
|  | Cardiorespiratory fitness (half-mile run) | 390.3 | 105.3 | 40 | 338.2 | 89.1 | 40 | 367.3 | 84.9 | 34 | 356.5 | 75.1 | 34 |
|  | Flexibility (SAR) | 6.5 | 4.9 | 40 | 6.7 | 5.3 | 40 | 4.6 | 5.6 | 34 | 5.9 | 6.1 | 34 |
| Tsang and DiPasquale, 2011 | Muscular strength (keen extension) | 86.88 | 16.93 | 11 | 77.18 | 12.34 | 11 | 79.53 | 18.12 | 14 | 69.98 | 14.83 | 14 |
| Ozen, 2012 | Body composition (body fat %) | 16.9 | 2.8 | 10 | 16.5 | 1.9 | 10 | 17.6 | 3.2 | 9 | 17.7 | 2.4 | 9 |
| Park et al., 2012 | Muscular strength (keen extension) | 19.2 | 6.5 | 16 | 26.3 | 7.9 | 16 | 24.4 | 8.7 | 15 | 26.7 | 7.4 | 15 |
| Chaouachi et al., 2014 EG1 | Muscular strength (leg press) | 66.16 | 22.13 | 14 | 83.45 | 24.06 | 14 | 72.02 | 22.45 | 7 | 77.68 | 23.60 | 7 |
| Chaouachi et al., 2014 EG2 | Muscular strength (leg press) | 76.89 | 28.03 | 14 | 95.91 | 27.50 | 14 | 72.02 | 22.45 | 7 | 77.68 | 23.60 | 7 |
| Racil et al., 2015 | Body composition (body fat %) | 41.7 | 3.6 | 26 | 38.8 | 3.7 | 26 | 39.3 | 1.7 | 23 | 36.5 | 1.3 | 23 |
|  | Body composition (BMI) | 2.9 | 0.3 | 26 | 2.6 | 0.2 | 26 | 2.9 | 0.2 | 23 | 2.4 | 0.3 | 23 |
|  | Body composition (lean mass) | 48.2 | 5.6 | 26 | 49.6 | 5.7 | 26 | 50.9 | 2.9 | 23 | 51.2 | 3.8 | 23 |
|  | Cardiorespiratory fitness (VO_2_max) | 36.0 | 1.7 | 26 | 39.4 | 1.8 | 26 | 36.7 | 1.1 | 23 | 39.2 | 1.0 | 23 |
| Marín-Cascales et al., 2015 | Muscular strength (knee extension) | 90.4 | 20.4 | 14 | 99.2 | 26.2 | 14 | 92.7 | 17.8 | 10 | 93.4 | 16.1 | 10 |
|  | Body composition (body fat%) | 41.8 | 4.4 | 14 | 41.1 | 4.1 | 14 | 40.4 | 4.7 | 10 | 42.1 | 4.6 | 10 |
|  | Body composition (lean mass) | 39.8 | 5.1 | 14 | 40.6 | 5.5 | 14 | 42.2 | 4.4 | 10 | 42.0 | 5.7 | 10 |
| Nobre et al., 2017 | Body composition (body fat%) | 35.4 | 6.3 | 40 | 35.9 | 6.3 | 40 | 40.1 | 0.5 | 19 | 38.5 | 0.6 | 19 |
|  | Body composition (BMI) | 23.8 | 2.53 | 40 | 23.6 | 2.53 | 40 | 24.8 | 3.92 | 19 | 24.9 | 3.35 | 19 |

| Marín-Cascales et al., 2017 | Muscular strength (knee extension) | 94.3 | 18.7 | 13 | 106.8 | 22.2 | 13 | 92.7 | 17.8 | 10 | 93.4 | 16.9 | 10 |
| --- | --- | --- | --- | --- | --- | --- | --- | --- | --- | --- | --- | --- | --- |
|  | Body composition (body fat%) | 42.2 | 4.2 | 13 | 41.2 | 3.3 | 13 | 40.4 | 4.7 | 10 | 41.3 | 5.1 | 10 |
|  | Body composition (lean mass) | 39.9 | 5.2 | 13 | 39.9 | 6.2 | 13 | 42.2 | 4.4 | 10 | 42.0 | 5.7 | 10 |
| Qi et al., 2019 | Body composition (BMI) | 18.07 | 3.28 | 31 | 18.94 | 0.65 | 31 | 19.97 | 4.73 | 15 | 19.03 | 0.66 | 15 |
|  | Body composition (lean mass) | 30.10 | 5.67 | 31 | 31.25 | 2.20 | 31 | 30.50 | 4.06 | 15 | 30.39 | 2.20 | 15 |
|  | Muscular strength (biceps curl) | 7.68 | 2.09 | 31 | 8.76 | 1.38 | 31 | 7.73 | 1.47 | 15 | 7.49 | 1.38 | 15 |
| Van Roie et al., 2020 | Muscular strength (leg press) | 175.7 | 50.87 | 14 | 211.8 | 53.18 | 14 | 162.1 | 50.87 | 14 | 161.7 | 52.79 | 14 |
| Radwan et al., 2021 | Flexibility (SAR) | 24.5 | 1.6 | 20 | 27.8 | 1.78 | 20 | 24.7 | 1.35 | 20 | 25.1 | 1.2 | 20 |
|  | Muscular strength (keen extension) | 37 | 4.2 | 20 | 42 | 2.8 | 20 | 35 | 3.3 | 20 | 38 | 4.1 | 20 |
| Singh et al., 2022 EG1 | Cardiorespiratory fitness  (Cooper test) | 2452 | 223 | 25 | 2610 | 224 | 25 | 2396 | 303 | 13 | 2403 | 282 | 13 |
| Singh et al., 2022 EG2 | Cardiorespiratory fitness  (Cooper test) | 2434 | 189 | 25 | 2678 | 132 | 25 | 2396 | 303 | 12 | 2403 | 282 | 12 |
| Marzouki et al., 2022 study 1 EG1 | Cardiorespiratory fitness (VO_2_max) | 42.4 | 2.8 | 20 | 46.0 | 2.8 | 20 | 42.0 | 3.0 | 10 | 42.6 | 3.0 | 10 |
| Marzouki et al., 2022 study 1 EG2 | Cardiorespiratory fitness (VO_2_max) | 42.1 | 2.8 | 20 | 46.0 | 2.6 | 20 | 42.0 | 3.0 | 10 | 42.6 | 3.0 | 10 |
| Marzouki et al., 2022 study 2 EG1 | Cardiorespiratory fitness (VO_2_max) | 42.4 | 3.1 | 20 | 46.1 | 3.4 | 20 | 42.0 | 3.3 | 10 | 42.7 | 3.2 | 10 |
| Marzouki et al., 2022 study 2 EG2 | Cardiorespiratory fitness (VO_2_max) | 42.3 | 2.7 | 20 | 46.1 | 3.0 | 20 |  | 3.3 | 10 | 42.7 | 3.2 | 10 |
| Bulqini et al., 2023 EG1 | Muscular strength (leg muscular strength) | 464.91 | 104.82 | 10 | 540.39 | 90.42 | 10 | 490.25 | 62.79 | 5 | 501.85 | 62.79 | 5 |
| Bulqini et al., 2023 EG2 | Muscular strength (leg muscular strength) | 482.55 | 57.87 | 10 | 619.97 | 43.98 | 10 | 490.25 | 62.79 | 5 | 501.85 | 62.79 | 5 |
| Witzke and Snow, 2000 | Muscular strength (keen extension) | 96.0 | 19.9 | 25 | 107.7 | 17.3 | 25 | 107.2 | 23.5 | 28 | 112.3 | 22.2 | 28 |
|  | Body composition (body fat %) | 24.3 | 7.9 | 25 | 24.3 | 7.0 | 25 | 24.1 | 3.9 | 28 | 23.3 | 5.7 | 28 |
|  | Body composition (lean mass) | 42.6 | 5.8 | 25 | 43.0 | 5.2 | 25 | 43.1 | 5.7 | 28 | 43.7 | 5.8 | 28 |
| Villada et al., 2016 EG1 | Body composition (BMI) | 26.78 | 3.88 | 15 | 25.64 | 2.71 | 15 | 25.17 | 2.61 | 8 | 27.58 | 2.79 | 8 |
|  | Body composition (body fat %) | 48.37 | 5.42 | 15 | 47.04 | 5.22 | 15 | 44.23 | 5.67 | 7 | 52.10 | 2.14 | 7 |
| Villada et al., 2016 EG2 | Body composition (BMI) | 27.52 | 5.26 | 15 | 25.65 | 4.16 | 15 | 25.17 | 2.61 | 7 | 27.58 | 2.79 | 7 |
|  | Body composition (body fat %) | 43.75 | 6.04 | 15 | 42.71 | 6.23 | 15 | 44.23 | 5.67 | 8 | 52.10 | 2.14 | 8 |
| Thaqi et al., 2020 | Body composition (MBI) | 20.63 | 3.6 | 110 | 20.97 | 3.20 | 110 | 20.95 | 3.79 | 110 | 21.21 | 3.64 | 110 |
|  | Muscular strength (push-ups) | 16.8 | 7.38 | 110 | 24.5 | 7.36 | 110 | 17.4 | 7.97 | 110 | 17.9 | 8.02 | 110 |
|  | Muscular endurance (sit-ups) | 19.0 | 3.78 | 110 | 26.6 | 3.85 | 110 | 19.9 | 4.24 | 110 | 20.3 | 4.17 | 110 |
| Almeida et al., 2021 | Muscular strength (handgrip) | 9.8 | 2.9 | 73 | 10.6 | 3.0 | 73 | 10.3 | 2.8 | 43 | 10.9 | 3.6 | 43 |
|  | Cardiorespiratory fitness (VO_2_max) | 46.3 | 1.7 | 73 | 46.6 | 3.0 | 73 | 46.6 | 2.2 | 43 | 46.4 | 2.4 | 43 |
|  | Flexibility (SAR) | 26.1 | 5.2 | 73 | 26.8 | 5.0 | 73 | 27.9 | 4.9 | 43 | 27.1 | 5.3 | 43 |
| **Other data format** | **Test** | **EG** | | | |  |  | **CG** | | | | |  |
|  |  | **Pre-**  **mean** | **Post-**  **mean** | **n** | **p** |  |  | **Pre-**  **mean** | **Post-mean** | **n** | **p** |  |  |
| Nobre et al., 2017 (53) | Muscular strength (handgrip) | 13.15 | 17.91 | 40 | 0.04 |  |  | 13.27 | 14.46 | 19 | 0.23 |  |  |
|  | Flexibility (SAR) | 22.45 | 28.43 | 40 | 0.05 |  |  | 25.49 | 26.86 | 19 | 0.24 |  |  |
|  | Cardiorespiratory fitness (VO_2_max) | 51.42 | 46.40 | 40 | 0.10 |  |  | 49.37 | 51.04 | 19 | 0.78 |  |  |

Note: EG, experimental group, CG, control group; SAR, sit and reach; VO2max, maximal oxygen consumption; MBI, body mass index; SD, standard deviation
